# FDG-PET in juvenile systemic lupus erythematosus

**DOI:** 10.1186/1546-0096-9-S1-P244

**Published:** 2011-09-14

**Authors:** M Alberdi, A Jørgensen, I Law, S Nielsen, MB Jørgensen

**Affiliations:** 1 Psychiatric Centre Copenhagen, Rigshospitalet, Copenhagen; 2 The PET and Cyclotron Unit, Department of Clinical Physiology and Nuclear Medicine, Rigshospitalet, Copenhagen; 3 Department of Pediatrics, Rigshospitalet, Copenhagen

## Introduction

Psychiatric manifestations in juvenile systemic lupus erythematosus (JSLE) are not uncommon, and they often pose diagnostic and therapeutic challenges. Magnetic resonance imaging (MRI) of the brain is often without abnormalities. Fluorodeoxyglucose positron emission tomography (FDG PET) detects relative reductions in regional cerebral glucose metabolism, and has been suggested as a more sensitive diagnostic tool. We describe two cases of JSLE with CNS involvement and distinct psychiatric symptoms, in which FDG PET showed, marked metabolic disturbances that normalized with successful treatment.

## Case1

A ten year old Asian girl, diagnosed with JSLE, based on the presence of exanthema, arthritis, vasculitis, nephritis, anemia and lymfopenia and positive for anti-nuclear (ANA) and anti-DNA antibodies. She was admitted due to recurrent episodes of psychosis with delusions and visual hallucinations, accompanied by severe anxiety, agitation, self mutilation and aggression. Rituximab in combination with various antipsychotics had little effect. There were no signs of epileptic activity. MRI with angio-sequence was normal. FDG PET showed hypermetabolism in the basal ganglia. Treatment with cyclophosphamide was initiated. Within six months of treatment, the severe CNS symptoms were gone. New FDG PET scans were normalized.

## Case2

A 16 year old Indian boy, with no known predisposition for mental illness, diagnosed with JSLE based on discoid lupus, anemia, arthralgia, nephritis and positive for ANA and anti-dsDNA antibodies. CNS symptoms began acutely with confusion, anxiety, incoherence and latency of speech. MRI with angio-sequence was normal. FDG PET showed mesial frontoparietal hypometabolism. Treatment with cyclophosphamide improved all but the CNS symptoms. Catatonia was diagnosed based on the presence of immobility, mutism, posturing, rigidity and waxy flexibility. After two months, decision was made to start electroconvulsive therapy. He showed clinical improvement almost immediately after the first of a total of 12 treatments. FDG PET showed complete recovery of the earlier findings.

## Conclusion

In these two cases of juvenile CNS SLE, FDG PET showed patterns of dysmetabolism which normalized with treatment, and thus provided an important alternative to MRI. FDG PET findings differed in association with differing symptomatologies, showing striatal hypermetabolism in the psychotic girl and frontoparietal hypometabolism in the catatonic boy.

We conclude that FDG PET should be considered in JSLE with CNS affection that ECT should be considered for catatonia in JSLE, after relevant anti-inflammatory treatment, and that further studies on the relation between CNS symptoms and FDG PET findings in JSLE is warranted.

